# Hypoglycemic and pancreatic protective effects of *Portulaca oleracea* extract in alloxan induced diabetic rats

**DOI:** 10.1186/s12906-016-1530-1

**Published:** 2017-01-11

**Authors:** Basma K. Ramadan, Mona F. Schaalan, Amina M. Tolba

**Affiliations:** 1 Department of Physiology, Faculty of Medicine for Girls (Cairo), Al-Azhar University, Cairo, Egypt; 2 Department of Biochemistry Faculty of Pharmacy, Misr International University, Km 28, Cairo-Ismailia road, Cairo PO Box 1, Heliopolis, Cairo Egypt; 3 Department of Anatomy, Faculty of Medicine for Girls (Cairo), Al-Azhar University, Cairo, Egypt

**Keywords:** Diabetes, IL-6, *Portulaca oleracea*, TNF-α, B-cell mass

## Abstract

**Background:**

Diabetes is a major public health concern. In spite of continuous new drug development to treat diabetes, herbal remedies remain a potential adjunct therapy to maintain better glycemic control while also imparting few side-effects. *Portulaca oleracea* has been traditionally used to manage several diseases due to the anti-oxidant and anti-atherogenic effects it imparts. To better understand the mechanisms associated with potential protective effect of *P. oleracea* extract against diabetes, alloxan-induced diabetic rats were used in this study.

**Methods:**

Forty Wistar rats (male, 7–8-wk-old, 140–160 g) were divided into four groups (*n* = 10/group): Group I (control), Group II (*P. oleracea*-treated; gavaged with *P. oleracea* extract daily [at 250 mg/kg] for 4 weeks), Group III (diabetic control; daily IP injection of alloxan [at 75 mg/kg] for 5 days) and Group IV (*P. oleracea*-pre-treated diabetic; gavaged with *P. oleracea* extract daily [at 250 mg/kg] for 4 weeks and then daily IP injection of alloxan [at 75 mg/kg] for 5 days). Body weight, food consumption, blood (serum) levels of glucose, C peptide, Hb A1C, insulin, tumor necrosis factor (TNF)-α and interleukin (IL)-6 were determined for all groups.

**Results:**

The results indicated that while Hb A1C, serum levels of glucose, TNF-α and IL-6 were all significantly decreased in the *P. oleracea-*pre-treated diabetic rats, these hosts also had significant increases in C peptide and insulin compared to levels in the counterpart diabetic rats. These results were confirmed by the histopathological assessments which showed marked improvement of the destructive effect on pancreatic islet cells induced by alloxan.

**Conclusion:**

P. oleracea extract is a general tissue protective and regeneartive agent, as evidenced by increasing β-cell mass and therefore improved the glucose metabolism. Thus, stimulation of *Portulaca oleracea* signaling in β- cells may be a novel therapeutic strategy for diabetes prevention.

## Background

Diabetes mellitus has become the third greatest “killer” after cancer and cardio-/cerebro-vascular diseases [1]. It is estimated that 5% of all deaths in the world are caused by diabetes, a number which will increase by 50% in the next 10 years [2]. At the time of clinical diagnosis, approximately 70% of the host’s total β-cell mass is destroyed as a consequence of immune-mediated processes [3]. As there are no certain cures for diabetes, an overarching goal in the treatment of all types of diabetes is preservation and even potential regeneration of β-cells.

Natural products isolated from medicinal plant sources have been used for the prevention and treatment of various diseases/pathologies, including cancers, heart disease, diabetes mellitus, and high blood pressure [4–6]. To date, more than 800 species have been investigated and their hypoglycemic effects reported [7].

*Portulaca oleracea*, (*P. oleracea*, Family *Portulacaceae*), also known as Purslane, is a herbaceous plant distributed throughout the world. It is eaten extensively around the Mediterranean and tropical Asian countries and has been used as a folk medicine in many countries. It contains many biologically active compounds and is a source of many nutrients including oxalic acid, alkaloids, ω-3 fatty acids, coumarins, flavonoids, cardiac glycosides, anthraquinones, linolenic acid, mono-terpene glycosides, *N*-trans-feruloyl tyramine, vitamins C and A, oleoresins-I and -II, saponins, tannins, saccharides, triterpenoids, and glutathione [8, 9]. The effects of *P. oleracea* extract have been evaluated in several model systems and is therefore listed as one of the most useful medicinal plants and named “Global Panacea” by the World Health Organization [10]. It is also rich in antioxidant vitamins and omega-3 fatty acids [11] and can be used for various curative purposes in health care especially in preventing some cardiovascular, inflammatory diseases and maintaining a healthy immune system [12, 13]. However, its usage for diabetes management was not rigorously evaluated, hence needs further elucidation. The studies reported in the literature were basically experimental observational studies, that did not provide enough mechanistic clarification for the hypoglycemic effect for *P. oleracea.*

The antidiabetic effect of the ethanolic extract of Purslane on high fat diet -induced diabetic rats was previously investigated by Hussein et al. [14]. They referred the antidiabetic effect to high content of flavonoids, phenolic compounds, melatonin and omega-3 fatty acids found in the ethanolic extract. However, other bioactive compounds found in purslane (dopamine, dopa, coumarins, alkaloids and saponins, polyphenols, flavonoids) may influence glucose metabolism by several mechanisms, such as inhibition of carbohydrate digestion and glucose absorption in the intestine, stimulation of insulin secretion from the pancreatic *ß*-cell, modulation of glucose release from liver, activation of insulin receptors and glucose uptake in the insulin sensitive tissues, and modulation of hepatic glucose output [14].

In another study of Lee et al. [15], the aqueous extract of P. oleracea was found to prevent diabetic vascular inflammation, hyperglycemia, and diabetic endothelial dysfunction in type II diabetic db/db mice, suggesting its protective role against diabetes and related vascular complications and renoprotective effect on diabetic nephropathy accelerated by renal fibrosis and inflammation in type 2 diabetic db/db mice [16]. The crude polysaccharide extract of this plant was also found to lower blood glucose and modulate the metabolism of blood lipids and glucose in alloxan- [17] as well as in STZ-induced diabetic mice [18], whilst decreasing the levels of total cholesterol, triglycerides, and fasting blood glucose in type II diabetic mice [19]. Gu et al. [20] compared the hypoglycemic and antioxidant activities of the fresh and dried P. oleracea L. in insulin-resistant HepG2 cells and streptozotocin-induced C57BL/6 J diabetic mice. Their results indicated that both fresh and dried *P. oleracea* extract possessed antidiabetic and antioxidant activities, besides stronger activity was observed in the fresh herb [20].

The present study sought to determine the possible protective and curative effects of *P. oleracea* extract against diabetes, with a specific focus on its potential regenerative effects on β-cells. For these analyses, a well-known model, i.e., the alloxan induced diabetic rat, was employed.

## Methods

### Animals

Wistar rats (40 male, 7–8-wk-old, 120–150 g) were obtained from the Abo Rawash Breeding Farm (Cairo, Egypt). All rats were housed (five rats/25 × 30 × 30 cm cage) under specific pathogen-free conditions in facilities maintained at 21–24 °C with a 40–60% relative humidity and 12 h light/dark cycle. All rats had *ad libitum* access to standard rodent chow and filtered water and were acclimated for 2 weeks prior to initiation of the experiment. All procedures were approved by the Animal Care Committee of Al Azhar University. The “Principles of laboratory animal care” were followed, as well as specific national laws where applicable.

For the experiment, rats were randomly allocated into four groups (*n* = 10/group).

- **Group I**: Control rats were kept on a balanced diet and to be intraperitoneally (IP)-injected with 3.7 ml/kg normal saline every two days for 4 weeks and then an additional daily IP injections of saline for a subsequent 5 days.
- **Group II**: Rats were to be gavaged with *P. oleracea* extract at a daily dose of 250 mg/kg for 4 weeks and then receive daily IP injections of saline for a subsequent 5 days.
- **Group III**: Rats were to be intraperitoneally (IP)-injected with 3.7 ml/kg normal saline every two days for 4 weeks and then made diabetic by daily IP injection of alloxan (at 75 mg/kg) for 5 days [21].
- **Group IV**: Rats were gavaged with *P. oleracea* extract daily (250 mg/kg) for 4 weeks and then receive daily IP injection of alloxan (at 75 mg/kg) for 5 days*.*

### Preparation of *P. oleracea* extract

The aerial parts of the fresh plant were collected from Al Aiat farms, Giza, Egypt. Samples were verified for taxonomy by the Pharmacognosy department coordinator, and a voucher specimen of the plant material has been placed in the herbarium of Al- Azhar University. For the extract, the parts (1000 g) were washed with water, cut into small pieces, dried at 40 °C in an oven and then powdered. The powder was decocted in purified boiling water (at a ratio of 1 g per 9 ml) for 30 min and then allowed to cool to room temperature. The materials were then filtered through filter paper. The filtrate volume (and mass) was measured before being aliquoted into glass bottles and concentrated in a rotary evaporator under reduced pressure at 55 °C and dried in a bath of warm water and stored at 2–8 °C. Based on the density, volumes of the extract (and needed dilution made with saline) could then be made for use in dosing the rats at the daily dose of 250 mg/kg [22]. The yield of the water extract of P. oleracea was approximately 22.8% of plant powder. For the gavages over the 4-weeks period, volumes of 4 ml were never exceeded.

### Chemical analysis

The total phenols were determined according to the method of Danial and Georg [23], while the total flavonoids were determined according to Zhisen et al. [24]. The fractionation of poly phenolic compounds were determined by HPLC according to the method of Goupy et al. [25], fractionation of flavonoids were determined by HPLC according to method of Merken and Beecher [26], while carotenoids were determined according to Wettestein [27].

### Induction of diabetes

Alloxan monohydrate has been used to induce experimental diabetes due to its selective destruction of pancreatic islet cells. Alloxan was obtained from Algomhoria Chemical Co. (Cairo, Egypt) and dissolved in normal saline at a concentration of 100 mg/ml alloxan solution [28] (at 75 mg/kg).

For the study, overnight fasted rats were injected IP (immediately after alloxan preparation) with the alloxan at a dose of 75 mg/kg, and then administered 50% dextrose-saline subcutaneously within 12 h after each alloxan injection to minimize/prevent mortality [29].

### Blood glucose monitoring

At the end of the experiment the blood glucose level of all groups was monitored after 8 h of fasting conditions. The samples obtained from the tail vein using a digital glucometer (Accu-chek® Advantage, Roche Diagnostic, Mannheim, Germany). Rats with blood glucose levels > 200 mg/dl were considered diabetic.

### Blood sampling

After12 h over night fasting, morning blood samples were collected from retro-orbital venous plexus by capillary tubes under light ether anesthesia, at the end of the experimental period and before animal scarification for pancreatic sampling. Blood was separated into two aliquots; one was anti-coagulated for HbA1c assessment and the other for serum separation. The first aliquot was heparinized and glycosylated hemoglobin Hb A1c has been defined operationally as the fast fraction hemoglobins HbA1 (HbA1a, A1b, A1c) that elute first during column chromatography.

The second aliquot was allowed to clot at room temperature and then centrifuged at 3000 rpm for 15 min; resultant sera were stored at −80 °C until assayed. The sera were eventually analyzed for C peptide (using a quantitative immuno-enzymatic colorimetric method using NOVATEC IMMUNDIAGNOSTICA GMBH, Germany cat number. DNOV112 [30] and insulin using the method of Eastham [31]).

Serum levels of tumor necrosis factor (TNF)-α and interleukin (IL)-6 was determined using commercial ELISA kits (RayBio® Rat, RayBiotech, Norcross, GA, USA) according to manufacturer protocols. The level of sensitivity of the kit was less than 25 pg TNFα/ml and less than 30 pg IL-6/ml.

### Body weight and food consumption

Body weight was measured weekly and the food consumption measured daily.

### Pancreatic tissue sampling

At the end of the experimental period, the animals were euthanized under general anesthesia, a midline incision ≈ 4 cm in length was induced in the abdomen, and the pancreas dissected out. Half of the pancreatic tissue (including tail part) was fixed in 10% neutral formalin and subsequently embedded in paraffin. Tissue sections of 5 μm were then prepared and stained by hematoxylin and eosin (H&E). Other sections underwent Masson trichrome (MT) staining to confirm a presence of any fibrotic tissues and to assess blood vessels in the tissue. Immunohistochemical detection of proliferative activity of the pancreatic cells was performed by using anti-Ki67.

Morphometric assessment was done on (H&E) stained slides using the image analyzer optical micrometer (TS view), objective lens of magnification 10 and eye piece of magnification of 12.5 binuclear microscope. The number of islets per fixed square area of 11703.6 μm^2^ was counted. Average area of the islets was determined by measuring the area of 4 islets in each section and totally 20 islets in each group. All measurements for each group were averaged and these results were subjected to statistical analysis.

### Statistical analysis

All results were expressed as mean ± SEM. Statistical analyses were performed using a one-way analysis of variance [ANOVA] followed by a Bonferroni *post hoc* multiple comparison test. All analyses were performed using computer program SPSS [version.18]). A *p*-value < 0.05 was considered as statistically significant.

## Results

The chemical composition *of P. oleracea* was analysed to find the active ingredients in this plant. The aqueous extract showed that total acidity was 0.15%, and pH value was 4.9. The antioxidant components were found to include total phenol (3.2 mg/100 g), flavonoids (6.2 mg/100 g) total chlorophyll (280 mg/100 g) and total carotenoids (45. mg/100 g) (as shown in Table 1). The phenolic compound were fractionated to catechein, chlorogenic, salcylic and pyrogallol while flavonoids were fractionated to rosmarinic, rutin, quercitrin with their respective levels, as illustrated in Table 2.

**Table 1:** Chemical composition of aqueous extract of P.oleracea (on wet weight basis)

Components	Aqueous extract of P.oleracea
Total acidity%	0.15
pH	4.9
Total phenolic compounds (as gallic acid)	3.2
Total flavonoids (as quercetin)	6.2
Total chlorophyll	280
Total carotenoids	45

**Table 2:** Fractionation of aqueous extract of P. oleracea polyphenol and flavonoids components by HPLC (mg/100 g wet weight)

Components of aqueous extract	(mg/100 g wet weight)
Phenolic compounds	
Caffeic acid	0.17
p-coumaric acid	0.52
Apigenin	0.4
Chlorogenic acid	0.9
Salicylic acid	0.8
Catechin	0.91
Flanonoids	
Rosmarinic acid	2.9
Rutin	0.1
Quercetin	0.3

### Effect of Portulaca Oleracea (P. Oleracea) on body weight and food intake (Table 3)

**Table 3:** Effect of *P. oleracea* extract on body weight and food intake

Parameter	Group I (Control)	Group II (PO-Control)	Group III (Diabetes)	Group IV (PO-Diabetic)
Body weight (g) at the beginning of experimental period	200.4 ± 12.9	^a^195.4 ± 11.5	^a^170.4 ± 10.5	^a^165.0 ± 11.8
Body weight (g) at the end of experimental period	210.4 ± 9.9	^a^180.4 ± 10.5	^a^165.0 ± 10.8	^b^202.1 ± 13.8
Food intake (g/d) at the beginning of experimental period	60.2 ± 1.0	^a^61.5 ± 0.5	^a^80.5 ± 0.9	^b^62.2 ± 0.7

Values are presented as means ± SEM

^a^Value significantly different vs. PO-control group or ^b^from diabetic group, at *P* < 0.05

The present study showed a significant decrease in the body weight in control rats that received P. Oleracea extract (Group II) (*P* < 0.05) from 195.4 ± 11.5gm to 180.4 ± 10.5 gm, when compared to control rats after four weeks of the experimental period. The induction of diabetes (group III) showed a significant reduction (*P* < 0.05) of body weight from 200.4 ± 12.9 gm to 165 ± 10.8gm, when compared to control rats. Moreover, the diabetic rats which were pretreated with P. Oleracea extract (group IV) were normalized (*P* < 0.05) from to 165 ± 11.8 gm to 202.10 ± 13.8, when compared to diabetic rats, while the decrease (*P* > 0.05) from 200.4 ± 12.9 gm to 210.4 ± 9.9 gm was nonsignificant when compared to control group.

As regards to food intake, P. Oleracea extract supplementation to rats showed a nonsignificant (*P* > 0.05) increase (from 60.25 ± 1.04 gm to 61.50 ± 0.54 gm), when compared to control rats, whereas diabetic group showed a significant increase of food intake (*P* < 0.05) from 60.25 ± 1.04 gm to 80.50 ± 0.94 gm, when compared to control rats. Moreover, the diabetic rats pretreated with P. Oleracea extract showed nonsignificant increase (*P* > 0.05) from 60.25 ± 1.04 gm to 62.25 ± 0.7 gm, when compared to control group but showed a significant reduction from 80.50 ± 0.94 gm to 62.25 ± 0.7 gm, when compared to diabetic rats.

### Effect of P. Oleracea extract on glucose homeostasis indicators (glucose, C peptide, Insulin) as well as inflammatory markers (TNF-α and IL-6) (Table 4)

**Table 4:** Effect of treatments on blood glucose, HbA1c, serum C-peptide, insulin, IL-6 and TNF−α

	Group I (Control)	Group II (PO-Control)	Group III (Diabetic)	Group IV (PO-diabetic)
Blood Glucose	119.8 ± 1.0	109.0 ± 1.3	^a^293.2 ± 2.4	^a,b^125.0 ± 1.3
C peptide (ng/mL)	0.97 ± 0.03	1.00 ± 0.04	^a,^0.48 ± 0.02	^b^0.94 ± 0.02
Hb A1c (%)	4.99 ± 0.06	5.01 ± 0.07	^a^10.35 ± 0.12	^a,b^6.97 ± 0.02
Insulin (mIU/L)	35.50 ± 0.09	36.02 ± 0.08	^a^18.97 ± 0.09	^b^33.50 ± 0.08
TNFa (pg/ml)	34.90 ± 0.17	34.77 ± 0.21	^a^49.70 ± 0.55	^b^35.20 ± 0.24
IL- 6 (pg/ml)	15.02 ± 0.13	14.91 ± 0.12	^a^28.90 ± 0.34	^b^16.26 ± 0.17

Values are presented as means ± SEM

^a^Value significantly different vs. PO- control group or ^b^from diabetic group, at *P* < 0.05

P. Oleracea extract supplementation to control rats (group II) for one week showed a non significant difference in the assessed parameters (blood glucose, C peptide, Insulin, TNF-α and IL-6), when compared to control rats. Therefore, all experimental groups were compared to P. Oleracea-control only. The induction of diabetes showed a significant increase (*p* < 0.05) in blood glucose level from 109.0 ± 1.3 mg/dl to 293.2 ± 2.4 mg/dl, when compared to PO- control rats. Pretreatment of diabetic rats with P. Oleracea extract for 4 weeks showed a significant reduction (*P* < 0.05) of blood glucose level from 293.2 ± 2.39 mg/dl to 125.0 ± 1.3 mg/dl when compared to diabetic rats.

P. Oleracea extract supplementation to control rats (group II) for four weeks showed nonsignificant increase (*P* < 0.05) in serum C peptide level from 0.97 ± 0.03 to 1.00 ± 0.04 when compared to control rats, whereas diabetic rats showed a significant decrease (*P* < 0.05) from 1.00 ± 0.04 to 0.48 ± 0.02 when compared to P. Oleracea - control rats. This drastic decrease was significantly reversed to control levels in the P. Oleracea-diabetic rats (from 0.48 ± 0.02 to 0.94 ± 0.02), when compared to diabetic rats.

As regards to serum HbA1c, the diabetic group showed a significant increase of serum HbA1c (*P* < 0.05) from 5.01 ± 0.07 to 10.35 ± 0.12, when compared to P. Oleracea-control rats. The latter high perecentage of HbA1c was significantly reduced to 6.97 ± 0.02 in P. Oleracea- diabetic rats, which remained still significantly higher than the P. Oleracea- control group.

In comparison to P. Oleracea - control group, the serum insulin level in the diabetic group showed a significant decrease (*P* < 0.05) from 36.02 ± 0.08 to 18.97 ± 0.09. This significant decrease was successfully reversed in the diabetic rats upon pretreatment with P. Oleracea extract for 4 weeks, as it showed a significant increase (*P* < 0.05) of serum insulin level from 18.97 ± 0.09 to 33.5 ± 0.08 when compared to diabetic rats.

As for the effect on TNF-α, the diabetic rats showed a significant elevation in the TNF-α levels (*P* < 0.05) from 34.77 ± 0.21 pg/ml to 49.70 ± 0.55 pg/ml when compared to control rats. The P. Oleracea- diabetic rats showed a significant decrease in TNF-α level from 49.70 ± 0.55 pg/ml to 35.2 ± 0.24 pg/ml, when compared to diabetic rats; this corrected level was nonsignificant from P. Oleracea -control levels.

Concerning the effect on IL-6, the diabetic group showed a significant increase from 14.91 ± 0.12 pg/ml to 28.9 ± 0.34 pg/ml, an elevation that was significantly decreased to 16.26 ± 0.17 pg/ml upon pretreatment with P. Oleracea (*P* < 0.05).

### Histopathological results

**Fig. 1:**
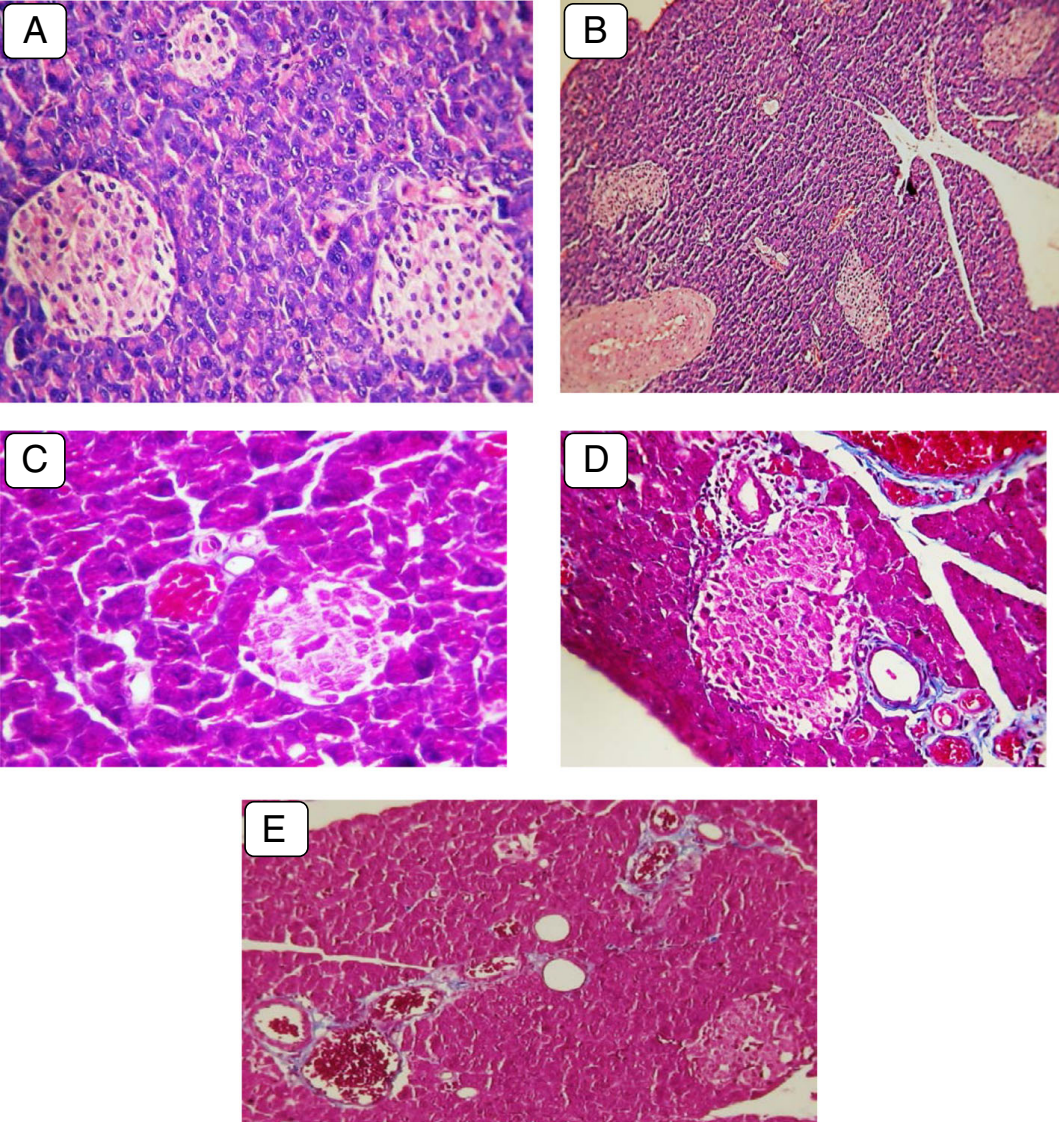
**a**: showing normal pancreatic tissue (H&E ×125). **b**: Tissue showing arteriosclerosis of medium-sized blood vessel and its branches in diabetic rat. **c**: Tissue with severe atrophy, depletion of cells, and necrosis in pancreatic islet of diabetic rat. **d**: Tissue with cell vacuolation and perivascular dropout and decreased vasculature among pancreatic islets, along with cellular infiltration by mononuclear cells in exocrine tissue in diabetic rat (Masson trichrome (MT) staining; 40× magnification). **e**: Tissue showing congested dilated blood vessels in diabetic rat (MT staining; 400× magnification)

Microscopic examination of pancreatic tissues of diabetic group showed variable sized pancreatic islets with arteriosclerosis (Fig. 1b), atrophy and reduction of its cellular components with areas of necrosis (Fig. 1c) compared to normal patterns (Fig. 1a). Some pancreatic tissues showed vacuolation and decrease vasculature with cellular infiltration with cells as lymphocytes and mononuclear cells with focal areas of degeneration, congestion and necrosis (Figs. 1d and e).

**Fig. 2:**
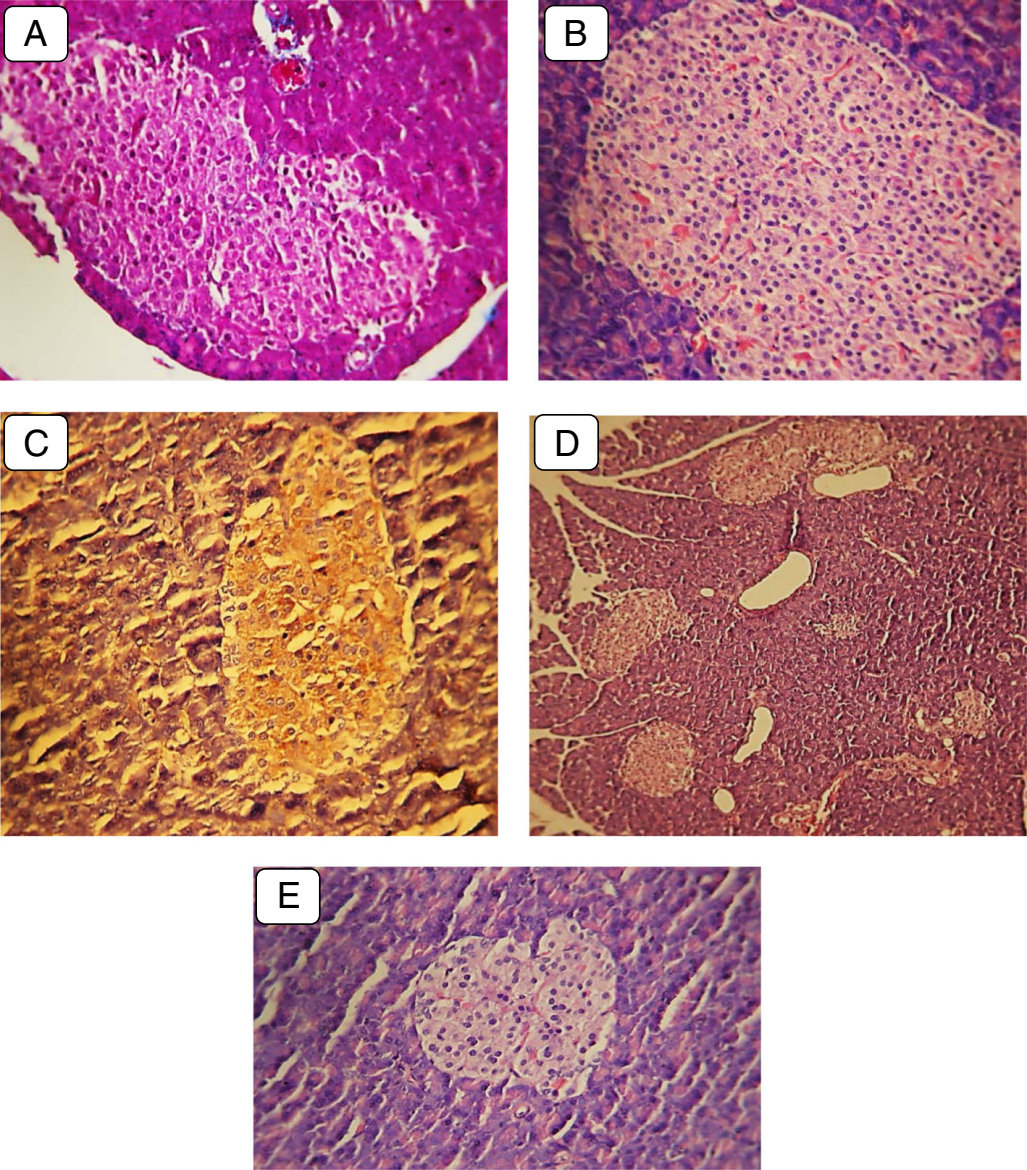
**a**: PO extract treated rats, showing increased vasculature in pancreatic islets to levels reaching near those seen in normal rat (H&E stain; 400× magnification). **b**: high power view, showing hypertrophy and hypercellularity of regenerating pancreatic islets (H&E×400). **c**: PO pretreated diabetic case showing high number and proliferative activity of pancreatic cells (Ki 67 immunostaining original magnification ×400). **d**: PO pretreated diabetic case showing hypercellularity of regenerating pancreatic islets. (H&E original magnification ×125). **e**: PO pretreated diabetic rats showing increased number of regenerating pancreatic islets (H&E ×125)

Pre-treatment with the extract for four weeks prior to induction of diabetes resulted in pancreatic morphologies resembling those of normal rats. Figure 2 panel describes the ameliorative process of β-cells after P. Oleracea extract administration in diabetic rats; increased vasculature in pancreatic islets is shown in Fig. 2a, reaching near-normal morphology of pancreatic islets as seen in Fig. 2b. Figures 2c and d illustrate the hypertrophy and hyper-cellularity along with increased blood vessel presence in the pancreas of P. Oleracea extract pre-treated rats. Similarly, there was a high level of proliferative activity among pancreatic islet cells as indicated by the increased levels of Ki-67 immunostaining (Fig. 2e).

The morphometric analysis, illustrated in Table 5, confirms the histological findings and reflects further the ameliorative impact of P. Oleracea extract on the viable pancreatic islets number and areas. It shows nonsignificant difference between control and P. Oleracea-control in terms of mean number of pancreatic islets and mean average of pancreatic islets area. As for the diabetic group it showed a significant decrease in mean number of islets (25.8%) as well as mean average of islets areas (47.5%), compared to P. Oleracea -control group. Interestingly, these decreases were significantly elevated, upon pretreatment with P. Oleracea for 4 weeks, to reach levels higher than the control levels.

**Table 5:** The morphometric analysis in experimental groups reflecting the pancreatic islets number and areas

	Group I (Control)	Group II (PO –Control)	Group III (Diabetic group)	Group IV (PO-diabetic)
The mean number of Pancreatic islets	2.9 ± 0.07	3.1 ± 0.06	^a^2.3 ± 0.08	^a,b^3.9 ± 0.09
The mean average of pancreatic islets area	159.9 μm ± 3.3	162.3 μm ± 3.5	^a^85.1 μm ± 3.4	^a,b^180.5 μm ± 4.8

Values are presented as means ± SEM

^a^Value significantly different vs. PO- control group or ^b^from diabetic group, at *P* < 0.05

## Discussion

Irrespective of the type of diabetes, β-cell mass preservation and/or increase are known to be important targets in management of diabetes as long as it reduces chronic microvascular complications in the eyes, kidneys and nerves [32].

The current study showed significant increases in blood glucose levels after alloxan injection of the rats. Etuk and Muhammed [33] and Adeyi er al. [34] attributed this increase in glucose levels to the reactive oxygen species induced by alloxan; this, in conjunction with a simultaneous massive increase in cytosolic calcium concentrations led to rapid destruction of pancreatic islet cells and a concomitant reduction in synthesis/release of insulin. This was confirmed here by histopathological results in alloxan-treated rats, as there were marked reduction in the size of cellular components of the islet cells along with variable levels of degeneration and the appearance of apoptotic cells. Such outcomes were in line with those of Adeyemi et al. [35] who noted also a significant reduction in the numerical density of islet cells (number/pancreas), islet cell area and diameter and β-cell density, in diabetic rats.

Interestingly, the *P. oleracea* extract pretreatment to diabetic rats here resulted in significant decreases in blood glucose levels. The possible contributing mechanisms is hypothesized to be due to the potentiation of insulin secretion from β-islet cells and consequent enhanced transport of blood glucose to peripheral tissues. The ameliorative and preventive effect of P oleracea against pancreatic islet tissue atrophy and necrosis in diabetic rat was represented on the histological images showing increased vasculature in pancreatic islets to levels reaching near those seen in normal rat, added to hypertrophy and hyper-cellularity with increased blood vessel presence. These effects could rationalize the improved β-cell number and size and hence better insulin secretion and better glycemic control.

In the current study the chromatographic analysis of the aqueas extract revealed phenolic compound fractionated to catechein, chlorogenic, salcylic and pyrogalol while flavonoids were fractionated to rosmarinic, rutin, quercitrin; these results are in agreement to the results obtained by Naciye [36] and Abd El-Aziz et al. [37].

Elkhayat et al. [38] attributed the antihyperglycemic effect of *P. oleracea* chloroformic extract to its high concentration of polysaccharides that were able to modulate blood lipid metabolism and decrease blood glucose. Moreover, chromatographic fractionation of the chloroform extract of *Portulaca oleracea* L. growing in Egypt afforded a new clerodene diterpene portulene, in addition to the known compounds lupeol, β-sitosterol, and daucosterol, which were reported for the first time from this plant. In another study of Shen and Lu [39] they attributed the hypoglycemic and hypolipidemic effect of the aqueous extract of P. *oleracea* to the hyper-insulinemia induced by *P. oleracea*. Moreover, they rationalized the improvement of insulin resistance in rats with T2DM, due to the amelioration of lipid metabolism and decrease free fatty acids, effects that are induced by the components of P. *oleracea*; ω-3 unsaturated fatty acid 6, vitamin E, vitamin C, carotene, glutathione. In our current study, the increased number of Ki 67 islet cells, with compensatory hyperplesia and hypertrophy support these data and provide a plausible explanation to the apparent hypoglycemic efficacy of P.oleracea.

Measures of serum C-peptide has been widely used as a marker of insulin secretion [40]. The present study revealed that induction of diabetes led to significant decrease in the levels of C- peptide and insulin when compared to control group. These results were consistent with those of Gabr et al. [41] who explained that this reduction was due to the destruction of the islet cells.

During diabetes, glycosylated hemoglobin is formed progressively and irreversibly over a period of time and is stable till the life of the RBC and is unaffected by diet, insulin or exercise on the day of testing [42]. Therefore, it is routinely used as a marker for long-term glycemic control [43]. The significant increase in HbA_1C_ levels in diabetic rats and its reversion in rats pre-treated with the extract; suggest that the overall blood glucose levels were being better controlled most likely due to improvements in insulin secretion in these hosts.

As there is a significant association between β-cell damage and inflammation, this study sought to assess the host circulating levels of pro-inflammatory cytokines TNF-α and IL-6 as they are known to play an important role in the pathogenesis/progression of insulin resistance [44, 45]. The present study showed elevated levels of serum TNF - α and IL-6 in the diabetic rats. These results were in agreement with Pennathur and Heinecke [46] and Mirza et al. [47]. Moreover, Sridevi et al. [48] reported that hyperglycemia leads to increased levels of monocytes that secrete increased amounts of TNF − α and IL-6 via up-regulation of protein kinase (PKC-α and PKC-β), P38 MAPK, and nuclear factor (NF)-κB.

Pretreatment with the aqueous extract of *Portulaca oleracea*, in the present study, induced significant reduction in serum levels of TNF-*α* and IL-6. These results were supported by the study of Lee et al. [49] who stated that pretreatment with the aqueous extract of *Portulaca oleracea*, inhibits TNF-*α*- induced production of intracellular reactive oxygen species (ROS) and over-expression of intercellular adhesion molecule-1 (ICAM-1), vascular cell adhesion molecule (VCAM)-1, and E-selectin in human umbilical vein endothelial cells (HUVECs) in a dose-dependent manner.

These results were also supported by the study of Xiao et al. [50] who reported that *P. oleracea* has a specific protective effect on damaged adipose cell induced by hyperlipdemic conditions. They reported the efficacy of *P. oleracea* to increase the cell viability and improve dyslipidemia with different degrees, through antiinflammmtory effect, hence it lowers the levels of TNF- *α* and IL-6 that adipose cell secretes. A similar ameliorative effect was provided by the study of Oshaghi et al. [51], who tested the aqueous extract of Anethum Graveolens L. in diabetic rats and proved its potential antidiabetic, antioxidant and antiglycation effects.

The body weight is a sensitive indicator that reflects the state of health of experimental animals and the decrease in body weight correlates with defects in body metabolism [52]*.* The expected reduction of body weight after alloxan injection was in agreement with Hassan and Emam. [53] and Ojo et al. [54] who attributed this reduction to the amelioration of hyperglycemia. The increase in the blood glucose resulting from the defective cellular uptake of glucose, forces the cells to utilize amino acids and fatty acids as a source of energy which eventually leads to the reduction of fats and tissue proteins which normally represents about 30 to 40% of total body weight. Thus, the excessive breakdown of tissue proteins due to diminished insulin response as well as the unavailability of carbohydrate for energy metabolism in diabetes mellitus results in decreased body weight. Interestingly, *P oleracea* administration to control rats caused a significant reduction in body weight, an effect that was resembled in the experimental research of Bai et al. [55] as well as clinical study of Esmaillzadeh et al. [56].

Regarding to the food intake, alloxan injection to normal rats in this study showed marked hyperphagia which may be attributed to the hyperglycemia. Guyton and Hall [57] explained this hyperphagia on physiological basis that the decrease in blood glucose concentration causes hunger, which has led to the so called glucostatic theory of hunger and feeding regulation. The satiety center are sensitive to arterio-venous gradient of blood glucose level, so high arterio-venous blood glucose gradient stimulates the satiety center and inhibits the feeding center inducing anorexia. In diabetes, although the blood glucose level is high, polyphagia is increased because the arterio-venous gradient is low as the cells cannot use the glucose due to absence of insulin.

## Conclusion

*Portulaca oleracea* is a general tissue protective and regenerative agent, as evidenced by increasing β cell mass and therefore improved the glucose metabolism. Thus, stimulation of *P. oleracea* signaling in β- cells may be a novel therapeutic strategy for diabetes prevention.

## Abbreviations

HUVEC: Human umbilical vein endothelial cells
ICAM-1: Intercellular adhesion molecule-1
IL-6: Interleukin-6
NF-κB: Nuclear factor-κB
PKC-α: Protein kinase α
PKC-β: Protein kinase β​
PO; *P. oleracea*: *Portulaca oleracea*
ROS: Reactive oxygen species
STZ: Streptozotocin
TNFα: Tumor necrosis factor
VCAM −1: Vascular cell adhesion molecule
